# Supplementary material for: The causal relationship between the human gut microbiota and pyogenic arthritis: a Mendelian randomization study
Source: Front Cell Infect Microbiol. 2024 Nov 26;14:1452480. doi: 10.3389/fcimb.2024.1452480 (PMC11629706; doi:10.3389/fcimb.2024.1452480)
Supplement: Supplementary file 1 [file DataSheet1.zip › mendelian test/ebi-a-GCST90027502.csv_forest.pdf]

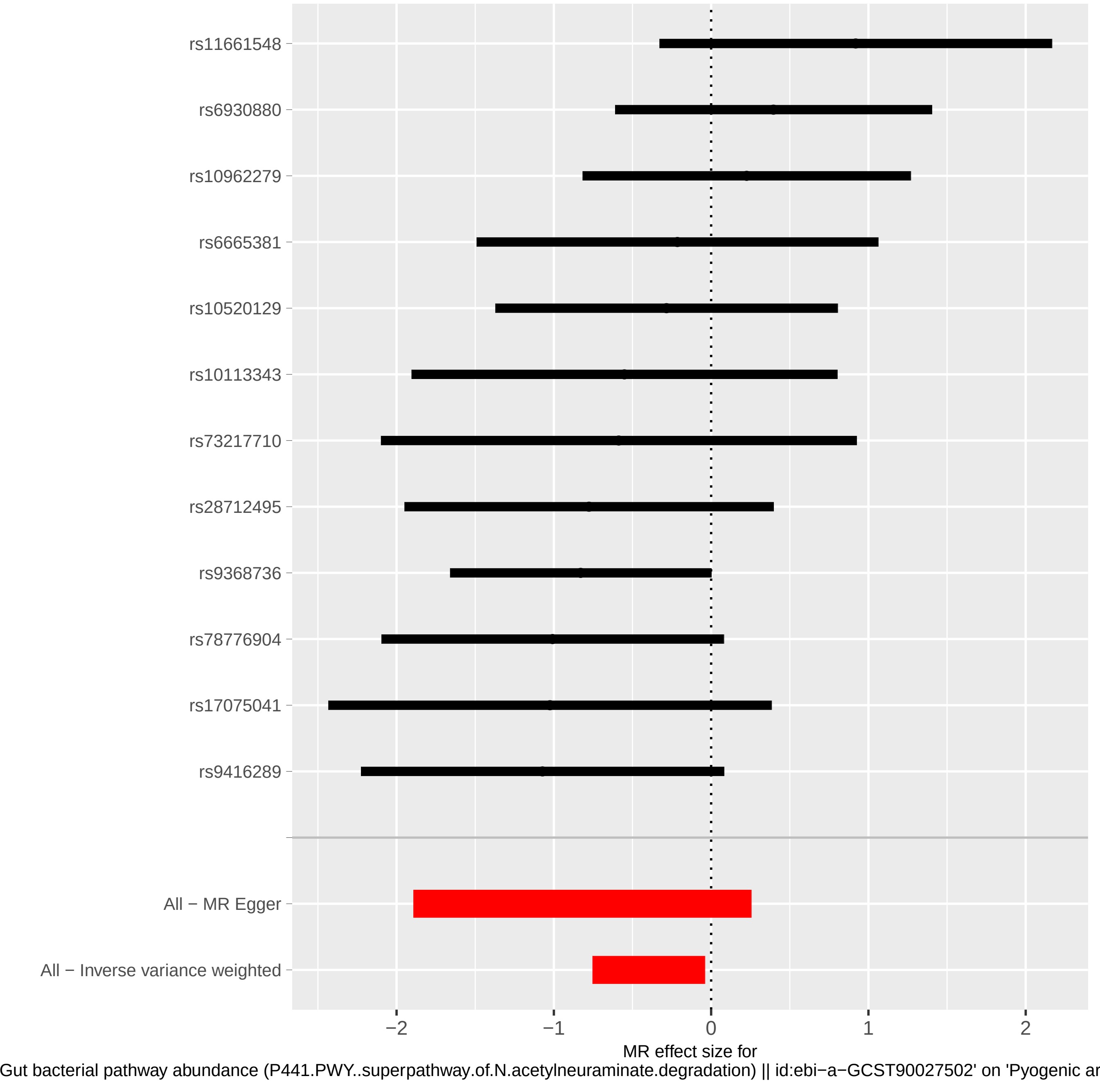
rs11661548
rs6930880
rs10962279
rs6665381
rs10520129
rs10113343
rs73217710
rs28712495
rs9368736
rs78776904
rs17075041
rs9416289
All − MR Egger
All − Inverse variance weighted
−2
−1
0
1
2
MR effect size for
Gut bacterial pathway abundance (P441.PWY..superpathway.of.N.acetylneuraminate.degradation) || id:ebi−a−GCST90027502' on 'Pyogenic ar
